# Near-Real Time Monitoring of Active Volcanoes from Space Using SLSTR (Sea and Land Surface Temperature Radiometer) SWIR (Shortwave Infrared) Observations

**DOI:** 10.3390/s26134262

**Published:** 2026-07-04

**Authors:** Carolina Filizzola, Giuseppe Mazzeo, Nicola Genzano, Carla Pietrapertosa, Francesco Marchese

**Affiliations:** 1 Istituto di Metodologie Integrate per l’Osservazione della Terra (IMIOT), Consiglio Nazionale delle Ricerche (CNR), c/da S. Loja, 85050 Tito, PZ, Italy; carolina.filizzola@cnr.it (C.F.); carla.pietrapertosa@cnr.it (C.P.); francesco.marchese@cnr.it (F.M.); 2 Department of Architecture, Built Environment and Construction Engineering (DABC), Politecnico di Milano, Via Ponzio 31, 20133 Milan, MI, Italy; nicola.genzano@polimi.it

**Keywords:** SLSTR, SWIR, volcanoes, NHI

## Abstract

**Highlights:**

**What are the main findings?**

- The NHI-SLSTR system for the NRT monitoring of thermal volcanic activity is presented.
- Eruptive activities of August–October 2025 are deeply investigated.

**What are the implications of the main findings?**

- Results show the capacity of the system in detecting and quantifying high-temperature volcanic features.
- The study confirms the complementary role of SWIR observations in active volcanoes monitoring.

**Abstract:**

The Sea and Land Surface Temperature Radiometer (SLSTR) is a dual-view scanning radiometer onboard the Sentinel-3A and Sentinel-3B satellites. This sensor provides data from the visible to the thermal infrared, with a temporal resolution of approximately 12 h. In this work, we present an automated system using shortwave infrared (SWIR) bands at 500 m spatial resolution to monitor active volcanoes in near real time. The system implements a normalized hotspot index (NHI) to detect and characterize high-temperature volcanic features in daylight and nighttime conditions. During the first three months of operation (i.e., August–October 2025), the system successfully identified several eruptive activities, with a false positive rate around 2.0%. The latter includes also true hot pixels associated with vegetation fires and other high-temperature sources. Results were assessed through comparison with the Fire Information for Resource Management System (FIRMS), the Middle Infrared Observations of Volcanic Activity (MIROVA), MODVOLC, and the S3-L2 FRP product. The preliminary comparison with the MIROVA-MODIS dataset reveals a good correlation in the estimates of fire radiative power over Etna (Italy) and Kilauea (Hawaii, USA), although discrepancies in the magnitude of this parameter remain significant also because of the SWIR retrieval method, which was optimized for gas flares. Despite the impact of snow-covered surfaces and band co-registration on the accuracy of hotspot detection, this study shows that the NHI-SLSTR system may provide a relevant contribution to the surveillance of active volcanoes from space, integrating information from other systems performing globally.

## 1. Introduction

For more than a decade, MODIS (Moderate Resolution Imaging Spectroradiometer), aboard NASA (National Aeronautics and Space Administration)-Terra and Aqua satellites, has represented a key instrument for the monitoring of thermal volcanic activity (e.g., [1,2,3,4,5,6,7,8,9]). This sensor offers a good compromise between spatial and temporal resolution (1 km at the near-nadir view, four observations per day) and some spectral channels in the MIR (medium infrared) and TIR (thermal infrared) bands, which were efficiently used to detect and characterize volcanic thermal features (e.g., [2,3]). Among those bands, the MIR channel, having a saturation temperature up to 500 K, has significantly improved estimates of the heat flux from intense lava effusions (e.g., [10]).

MODVOLC is a MODIS-based system specifically developed for the monitoring of active volcanoes on a global scale from space [1]. This system delivers hotspot products in terms of hot pixel number, MIR radiance, and radiant flux, which were largely used to investigate and quantify volcanic thermal emissions by satellite (e.g., [11]).

MIROVA (Middle InfraRed Observation of Volcanic Activity), which currently monitors more than 200 active volcanoes, has further improved the identification of volcanic thermal features (VTF) from space [6,12,13]. This system analyzes VIIRS (Visible Infrared Imaging Radiometer Suite) data to perform more accurate estimates of fire radiative power (FRP) [14], which is generally indicated as VRP (volcanic radiative power) when referring to volcanic activity (e.g., [15,16]). VIIRS flies aboard Suomi-NPP, JPSS-1 (NOAA-20), and JPSS-2 (NOAA-21) satellites, which guarantee at least six observations per day over the same regions [17], and offers a higher spatial resolution than MODIS (up to 375 m in the imaging bands), favoring the identification of small hotspots (e.g., [13]).

The NASA-FIRMS (Fire Information for Resource Management System) system performs the global hotspots monitoring through MODIS and VIIRS observations [18]. The FIRMS products are available within 3 h satellite overpass time and were widely used to investigate active volcanoes from space (e.g., [19,20]).

Other satellite-based systems developed for the identification and monitoring of volcanic hotspots use TIR data from ASTER (Advanced Spaceborne Thermal Emission and Reflection Radiometer, 90 m spatial resolution) to improve the identification of subtle hotspots using multi-temporal detection methods (e.g., [21]) and deep learning models (e.g., [22]). Although ASTER has a nominal temporal resolution of 16 days at the equator, the Urgent Request Protocol (URP) improves this resolution over some volcanoes by triggering additional observations, improving the temporal resolution to as low as 1–8 days [23].

While the above-mentioned systems mainly use MIR and/or TIR data from sensors at low spatial/high-temporal resolution (Table 1), the Google Earth Engine (GEE) Apps implementing the NHI (Normalized Hotspot Indices; [24]) algorithm enable the mapping of high-temperature volcanic features through the analysis of NIR (near infrared) and SWIR (shortwave infrared) radiances from OLI (Operational Land Imager) and MSI (Multispectral Instrument) sensors [25]. Indeed, the SWIR bands of these instruments are highly sensitive to active lava bodies, due to the peak of thermal emission shifting toward shorter wavelengths as temperature increases (e.g., [26]). On the other hand, while the SWIR bands of MSI and OLI are particularly suited for detecting hot targets, they are less sensitive to magmatic sources with temperatures below 500 K (e.g., [27]), which are better resolved in the MIR/TIR channels of MODIS and VIIRS.

By analyzing MSI and OLI data, the NHI tool allows users to perform time-series analyses in terms of hot pixels, total SWIR radiance and area covered by the hotspots. The NHI_MSI/OLI_ system then provides automated information about volcanoes with evidence of a thermal activity detected over the previous 48 h [28]. Both GEE-Apps leverage the advantages of the virtual Landsat-8/9 (L8/9) and Sentinel-2 (S2) constellation (i.e., global median average revisit interval of 2.7 days; [29]) to investigate and monitor active volcanoes through satellite data at mid-high spatial resolution.

The NHI algorithm was recently adapted to SWIR observations (500 m spatial resolution; see Table 1) from the SLSTR (Sea and Land Surface Temperature Radiometer) instrument aboard Sentinel-3A/3B satellites to investigate the Home Reef (Tonga) 2022–2024 eruption [30]. Results encouraged us to extend monitoring to other volcanoes through SLSTR SWIR data, with the aim of complementing information from MODIS and VIIRS-based systems. Indeed, while machine learning models applied to SLSTR MIR observations were tested over specific volcanic areas of interest [31], the S3-SLSTR FRP product, although developed for gas flaring sources, detects and quantifies even wildfires and volcanic hotspots in the SWIR bands, but only at nighttime [32,33].

The NHI-SLSTR system (https://sites.google.com/view/nhi-tool/sentinel-3, accessed on 2 April 2026) provides information about volcanic thermal features from both daytime and nighttime SWIR data through a dedicated web interface and KML (Keyhole Markup Language) products.

In this work, we present this automated hotspot monitoring system and results retrieved during the first three months of operation (i.e., August–October 2025). The aim of this work is to assess the system’s capacity in detecting and quantifying, in terms of FRP, high-temperature volcanic features from space. The contribution of the NHI-SLSTR system to surveillance of active volcanoes from space is evaluated also through comparison with information provided by other well-established satellite-based systems (e.g., MIROVA, MODVOLC; FIRMS), performing the NRT monitoring of thermal volcanic activity from space through MODIS and VIIRS data at different spatial and temporal resolutions.

Results of hotspot detection (including the correct volcano attribution) and FRP estimation are analyzed and discussed in detail in the next sections.

## 2. Data and Methods

### 2.1. SLSTR Instrument and Data Products

The SLSTR instrument flies aboard the Sentinel-3A and Sentinel-3B satellites, providing data in nine spectral bands—ranging from visible/near-infrared (VNIR) to thermal infrared—via a dual-view (near-nadir and oblique) scanning geometry based on two independent scan mirrors rotating in opposite directions. For bands S4 (0.87 µm), S5 (1.6 µm), and S6 (2.25 µm), two detector element sets (stripe A and stripe B) acquire imagery simultaneously, producing two distinct images with different geolocation. Specifically, the B-stripe detectors view a physically different point on the ground. The two detector stripes for the SWIR channels were originally intended to enable time domain stripe integration to improve the signal-to-noise ratio [34], while the additional F-bands (F1 and F2) were designed to better detect high-temperature features, thanks to the extended dynamic range [34].

The SLSTR level 1B product (‘SL_1_RBT’) provides global, geo-located radiometric measurements (radiances and brightness temperatures) for each pixel in a regular image grid for each view and spectral channel. The NRT (Near Real Time) products are available at the pick-up point in less than 3 h and retained for about a month in the EUMDAC (EUMETSAT Data Access Client) archive [35]. The Non-Time Critical (NTC) products are available at the pick-up points in less than 30 days after acquisition and contain better computed parameters (e.g., precise orbit data). Small differences are expected between NRT and NTC products due to auxiliary data used in NTC as compared with NRT (e.g., consolidated orbit files, analysis ECMWF data rather than forecast ECMWF meteorological dataset [35]), but also due to some differences in data processing (e.g., re-gridding algorithm [36]). Notwithstanding these slight differences, the two products have the same absolute geolocation of each measurement position [35].

In this work, we downloaded three months of SLSTR data (August, September, and October 2025) from EUMETSAT website by using EUMDAC v.3.0.0. Then, we performed the correction of the S5 and S6 TOA radiances using the suggested adjustment factors [37].

### 2.2. NHI Algorithm Adapted to SLSTR SWIR Data

The NHI_SWIR_ and NHI_SWNIR_ indices, analyzing NIR and SWIR TOA (top of the atmosphere) radiances, were originally proposed to map high-temperature features through MSI and OLI data [24]. Based on their different behavior (e.g., [24]), pixels with positive values of one or both indices are considered “hot” by the standard NHI algorithm. Negative critical values of the NHI_SWIR_ can be, however, explored to better map very small high-temperature targets [38] or to detect active lava bodies through coarser spatial resolution sensors [39,40]. In this context, after a first assessment of volcanic hotspot detections performed at the Home Reef [30], we developed an automated processing chain to monitor active volcanoes in NRT through SLSTR SWIR data, using the NHI algorithm configuration detailed in Table 2. It is worth mentioning that although the NHI-SLSTR system performs globally using NRT data, the assessment of the first three months of operation was carried out using the NTC ones for the reasons discussed in Section 2.1. On the other hand, since the discrepancies between NRT and NTC products remain minimal, slight differences in the use of NTC rather than NRT data are expected.

An image window of 0.02 × 0.02 degrees, in latitude and longitude from the central geographic coordinates, is set as the default by the system to search for hotspots over the active volcanoes. Although this conservative choice sped up system operation, it may impact the accuracy of hotspots detection as shown in Section 3. The assessment of hotspot detections was then performed using the GVP (Global Volcanism Program) database of 2013 (1422 volcanoes in number) [41], even though at the time of writing this paper, the system ingests the latest GVP database of March 2026 [42].

Detected hotspots were then quantified in terms of FRP/VRP through the formulation described in detail in the next section.

### 2.3. FRP Computation

The FRP_SWIR_/VRP_SWIR_ is computed using the approach proposed for gas flares with a temperature range of 1600–2200 K [43,44,45]:

(1) $$FRP_{SWIR}=\frac{A\sigma }{10^{6}\;p{\;\tau }_{SWIR}}\left(L_{SWIR}-L_{b,SWIR}\right)\;[\mathrm{MW}]$$

where, p = 6.1 × 10^−9^ W m^−2^ sr^−1^ mm^−1^ K^−4^ [43], A is the pixel area [m^2^], depending on the satellite zenith view angle (as we used level L1B with re-gridded satellite measures, we assumed A to be always equal to 500 × 500 m^2^), s = 5.67 × 10^−8^ W m^−2^ K^−4^ (Stefan-Boltzmann constant), t_SWIR_ is the atmospheric transmission in the SLSTR S6 band. The terms L_SWIR_ and L_b,SWIR_ [mW m^−2^ sr^−1^ nm^−1^] are the spectral radiances of the lava pixel and the mean spectral radiance of the background in the S6 band, respectively. The FRP estimates for gas flaring (GF) sources derived from SWIR observations have been reported with an uncertainty of approximately ±6.3% when using observations made at 2.2 µm and ±13.5% when using observations at 1.6 µm [44]. Although this approach, which is used by the Sentinel 3-SLSTR Level-2 FRP product, was originally developed for gas flaring (GF) sources, it can be extended to wildfires and VTF. However, because these phenomena exhibit lower temperatures, the increase in retrieval uncertainty must be taken into consideration. In recent studies the NHI detections were compared with the above-mentioned FRP product, showing a very good agreement in quantifying California’s wildfires of January 2025 [46]. This agreement should then characterize also high-temperature volcanic features such as lava flows, with the NHI-SLSTR system performing estimates of FRP also in daytime.

**Figure 1:**
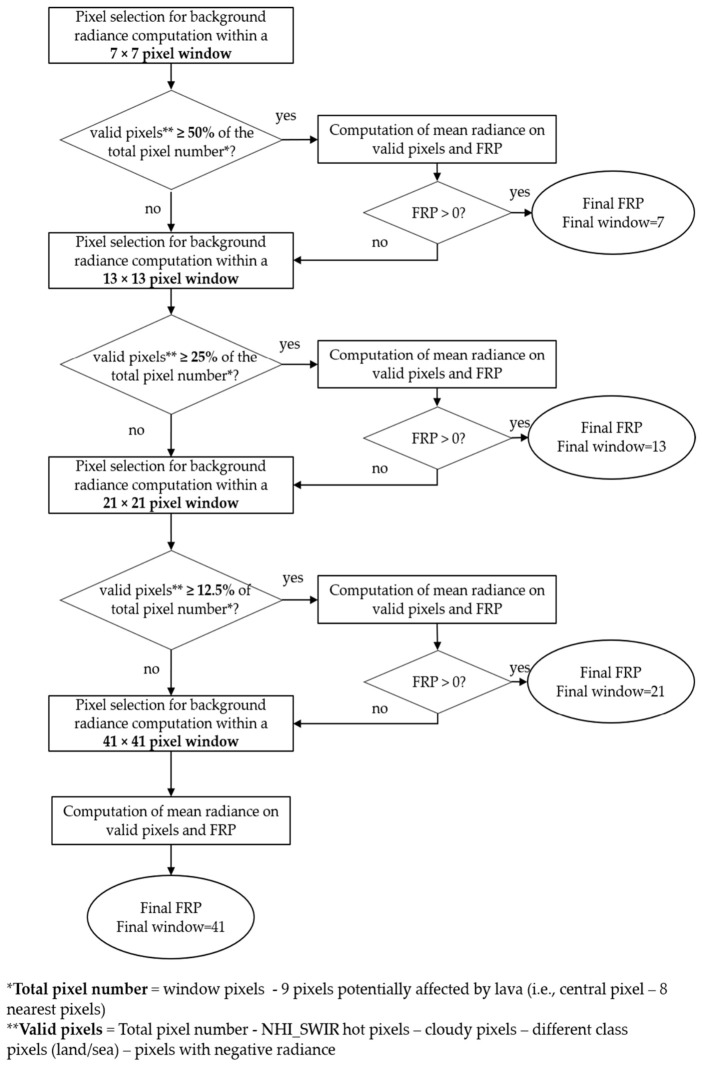
Flow diagram used for the computation of the background radiance (L_b_,_SWIR_) and the corresponding FRP value estimated using Equation (1).

Figure 1 shows the flowchart of the background radiance computation and the corresponding FRP, calculated according to Equation (1). The NHI-SLSTR system does not consider the 3 × 3 window size because the pixel radiance could potentially be affected by active lava bodies. Indeed, for all the windows used, the radiance of the central pixel and the eight nearest pixels has been excluded. The 5 × 5 size is not evaluated by the system due to the very small number of available pixels. Starting from a 7 × 7 window, the background radiance is automatically computed in a strict manner, using a percentage of valid pixels, which are halved as the window size (and consequently the number of available pixels) increases. The maximum window size used for the FRP computation (≈20 × 20 km^2^) is in line with indications about background representativeness to avoid false detections [47].

While positive FRP values indicate a proper background computation, invalid (i.e., negative) FRP values can be recorded when background radiance is greater than that of hot pixels (e.g., when the 41 × 41 image window is reached and a small number of non-representative pixels contributes to the background mean value). If there are no valid pixels to calculate the background radiance, the FRP is automatically set to −999. The background computation is performed also at nighttime (when the ambient SWIR radiance is close to zero) to take into consideration the instrumental noise.

Information on cloudy pixels and the land/sea mask is derived, for each satellite overpass, from ‘flags_an.nc’ (for stripe A) and ‘flags_bn.nc’ files (for stripe B) of the L1B SLSTR data. All bits from ‘cloud_an’ (for stripe A) and ‘cloud_bn’ (for stripe B) variables were considered to flag background pixels as cloudy or clear; 3rd (‘land’) and 4th (‘inland water’) bits from ‘confidence_an’ (for stripe A) and ‘confidence_bn’ (for stripe B) variables are combined to obtain a land-sea mask and classify background pixels as land or sea/water.

The t_SWIR_ value is computed using the following formulation [43]:

(2) $$\tau_{SWIR}=\exp\left(\frac{-t_{SWIR}}{\cos\left(A+Bq\vartheta_{v}+C{\left(q\vartheta_{v}\right)}^{2}\right)}\right)$$

where q is the constant p/180, t_SWIR_ is the atmospheric optical depth in the SWIR S6 channel, and ϑ_v_ is the satellite zenith view angle. The terms t_SWIR_, A, B, and C are linearly interpolated from the table available in the Sentinel-3 Optical Products and Algorithm Definition [43], depending on the total column water vapor (TCWV, kg/m^2^). Both ϑ_v_ and TCWV of the lava pixel are obtained for each SLSTR overpass from ‘geometry_tn.nc’ and ‘met_tx.nc’, respectively.

### 2.4. The NHI-SLSTR Web-Interface and KML Products

The NHI-SLSTR system provides automated information about high-temperature volcanic features in NRT (i.e., as soon as the NRT data are made available by EUMETSAT) through a dedicated web interface. The system makes freely available online also the KML files generated over the last 24 h. These files provide, separately for stripe A and stripe B, additional information at the pixel level, including satellite, which made the sensing, SWIR1 and SWIR2 radiances, size of the pixel window for background computation, atmospheric transmittance in the S6 band, and satellite zenith angle. The system currently does not store, however, any time-series of hotspot pixels or FRP over the monitored volcanoes.

**Figure 2:**
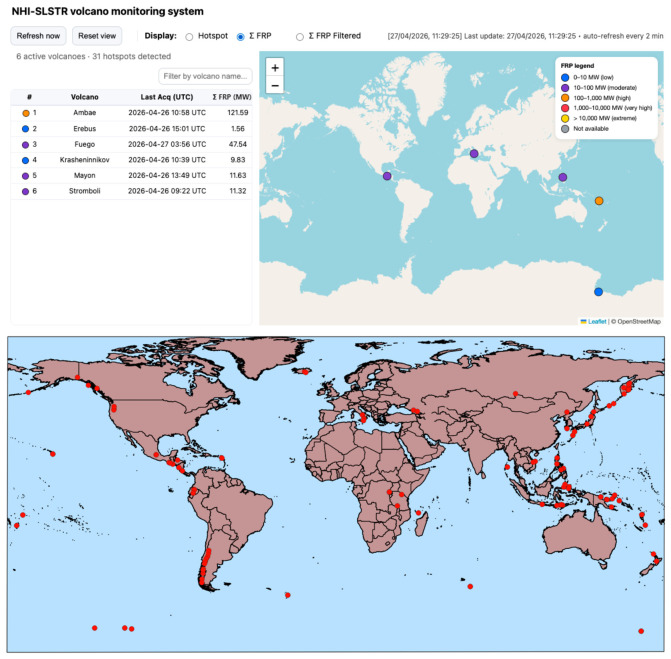
Web interface of the NHI-SLSTR monitoring system (https://sites.google.com/view/nhi-tool/sentinel-3, accessed on 30 June 2026). The upper panel shows the web interface of the NHI-SLSTR system, which provides information about active volcanoes in NRT, including hotspots detections and relative FRP values. The lower panel shows the global spatial distribution of the NHI-SLSTR hotspots detected during the first three months of operation (August–October 2025).

Figure 2 shows the web interface, together with the global spatial distribution of NHI-SLSTR detections of August–October 2025. The graphical interface displays the list of active volcanoes with evidence of an ongoing thermal activity in the hotspot section, detailing for each hot pixel the geographic coordinates, the satellite acquisition date and time, the NHI_SWIR_ and FRP values, and the relative confidence level (low/high). The latter takes into consideration the presence of snow-covered surfaces, which may represent a possible cause of false positives when negative critical values of the index are analyzed in daylight conditions (see next section), providing indications about the confidence level of detection.

Hot pixel centers may be directly visualized on a geographic map, providing information about the hot areas. The hot pixel centers are marked in different colors based on relative FRP range: 1–10 MW (blue; low); 10–100 MW (violet; moderate); 100–1000 MW (orange; high); 1000–10,000 (red; very high); >10,000 MW (extreme; yellow). Pixels with invalid FRP values are marked in grey. This color scale was chosen to better fit with information from the advanced NHI tool, which is under development.

In the “Σ FRP” section, the system details volcano-scale FRP values obtained by summing the fire radiative power of each detected hot pixel. In addition, the system also provides a “Σ FRP Filtered” section, in which the values are recalculated after filtering possible duplication effects through a clustering procedure based on a 300 m spatial threshold to reduce the possible hot pixel overestimation, which may occur, for example, between overlapping observations from the SLSTR A and B stripes. These different estimates of total FRP are assessed in Section 3.2.

## 3. Results

### 3.1. Hotspot Detection Assessment

During the period of August–October 2025, the NHI-SLSTR system automatically processed about 35,000 satellite imagery. Table 3 shows, for each month of operation, the number of analyzed satellite scenes and the hot pixels associated with thermal volcanic activity. Results were achieved without using any filtering procedure to take into consideration possible duplication effects.

Hotspot detections were assessed through: (a) GVP bulletins [48], verifying the occurrence of documented eruptive activities over the areas with detected hotspots; (b) visual inspection of SLSTR imagery (in the VNIR, SWIR, MIR, and TIR bands) to exclude false positives due to snow-covered surfaces and other factors; (c) information from the NHI tool (analyzing temporally close S2-MSI and L8/9 OLI/OLI2 observations) and the comparison with FIRMS, MODVOLC, MIROVA, and S3 SLSTR-L2 FRP datasets to confirm the occurrence of thermal volcanic activities unreported by GVP.

Results of the validation analysis are summarized in Table 4. The table shows that 93.7% of hot pixels flagged by the NHI-SLSTR system were correctly associated with VTF. Annex A1 reports details of those detections in terms of the dominant rock, the tectonic setting, and the country of the corresponding volcano.

About 3.0% of hot pixels were associated with uncorrected volcano attributions, about 0.5% with other high-temperature sources (vegetation fires and possible mining activities in geothermal areas), and only 2.8% with other factors causing false detections.

**Figure 3:**
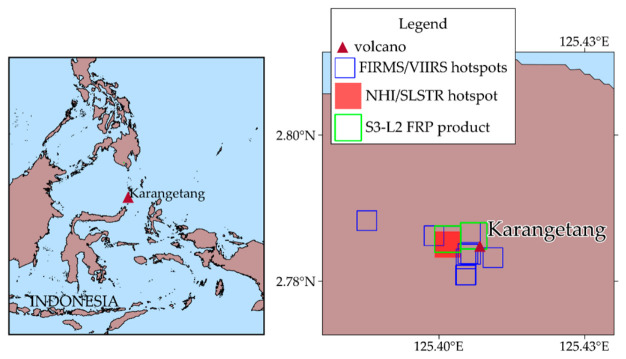
NHI-SLSTR system (stripe B; red areas) and S3 SLSTR-L2 FRP (stripe A; green areas) detections from S3B-SLSTR data of 30 September 2025 at 13:39 UTC showing hotspots detected over Karangetang (Indonesia) volcano (Indonesia). In blue, FIRMS detections from Suomi-NPP (16:56 UTC), NOAA-20 (04:38 UTC, 17:15 UTC), and NOAA-21 (16:28 UTC) VIIRS observations at 375 m spatial resolution. Coastlines from “Global Self-consistent, Hierarchical, High-resolution Shoreline Database_full (GSHHS_f)” [50].

**Figure 4:**
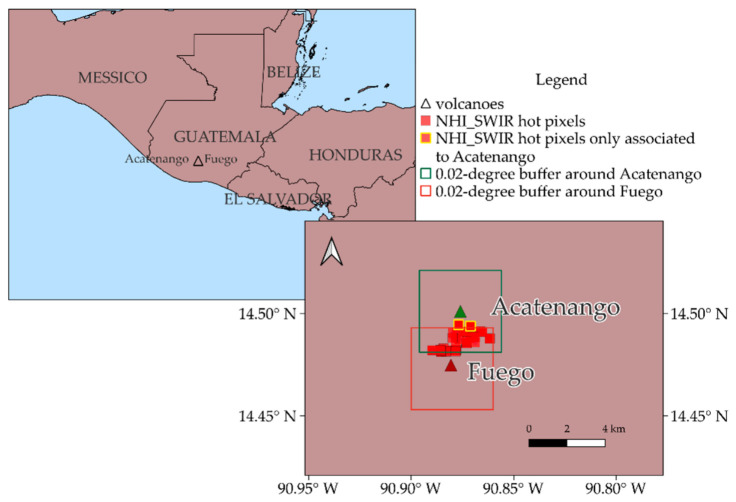
Hot pixels, overlapped on the geographic map of La Horqueta volcanic complex, erroneously attributed to the Acatenango (Guatemala), owing to buffer area (in green) of 0.02 × 0.02 degrees, set as default by the NHI-SLSTR system. Most of those pixels (~89%) were included also within the Fuego’s buffer (in red), while a few of them, in yellow, were outside the buffer area marked red. Coastlines are from GSHHS_f [50].

Among the hotspots correctly associated with thermal volcanic activity (96.7% in total, including those related to uncorrected volcano attribution), most of them were corroborated by GVP, while others were confirmed by the additional investigations detailed above (i.e., information from independent satellite-based systems). As an example, while the last Karangetang (Indonesia) eruption was reported by GVP on 5 September 2023, the NHI-SLSTR system identified a thermal anomaly over the S3-SLSTR image of 30 September 2025 at 13:39 UTC. This observation is consistent with FIRMS and S3 SLSTR-L2 FRP detections of the same day, as shown in Figure 3 (see areas marked in red, for comparison with green and blue ones). Table 4 also shows that several hot pixels, associated with documented eruptions, were erroneously attributed to spatially close quiescent/dormant volcanoes. This hotspot category includes the hot pixels erroneously flagged over Acatenango (Guatemala) and associated with lava flows from the Fuego volcano, belonging to the same volcanic complex (La Horqueta). This attribution error, ascribable to the buffer area set as default by the system, yielded the hot pixel duplication, as shown in Figure 4. A similar attribution error affected also the Kamen volcano, which is part of the Klyuchevskaya group, including Klyuchevskoy and Bezymianny [49].

Indeed, hot pixels erroneously attributed by the NHI-SLSTR system to the Kamen volcano should be related to lava flows from Klyuchevskoy [51,52] and possibly to strong degassing activity from Bezymianny. This volcano generated thermal anomalies, which were observed also by other satellite-based monitoring systems (e.g., [53]).

**Figure 5:**
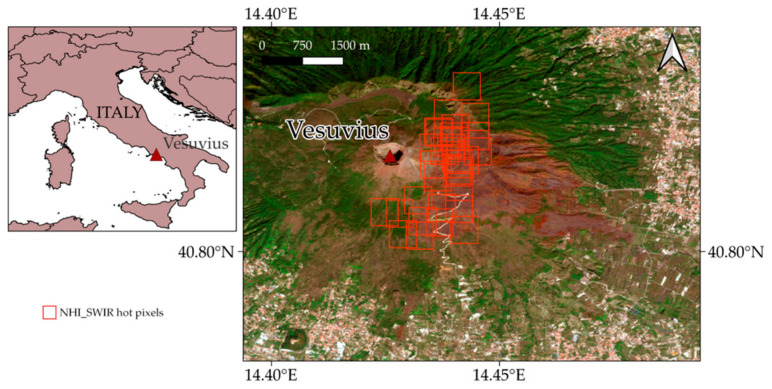
NHI-SLSTR detections (in red) of 8–10 August 2025 associated with an intense vegetation fire affecting the Vesuvius (Italy) volcanic area. Hot pixels were overlapped on the true color RGB (R: B4 = 665 nm, G: B3 = 560 nm; B: B2 = 490 nm) image from S2-MSI scene of 18 September 2025 at 09:50 UTC from Copernicus Browser https://browser.dataspace.copernicus.eu/ (accessed on 2 April 2026). The reddish-brown color on the eastern flank of the volcano indicates the burned area. Coastlines are from “GSHHS_f” [50].

Regarding the true hot pixels associated with wildfires, those shown in Figure 5 were detected over the Vesuvius (Italy) volcanic area, from S3-SLSTR SWIR data of 8–10 August 2025. These pixels were related to an intense forest fire, burning hundreds of hectares of vegetation, which lasted several days [54], activating the Copernicus Emergency Response Mechanism [55].

**Figure 6:**
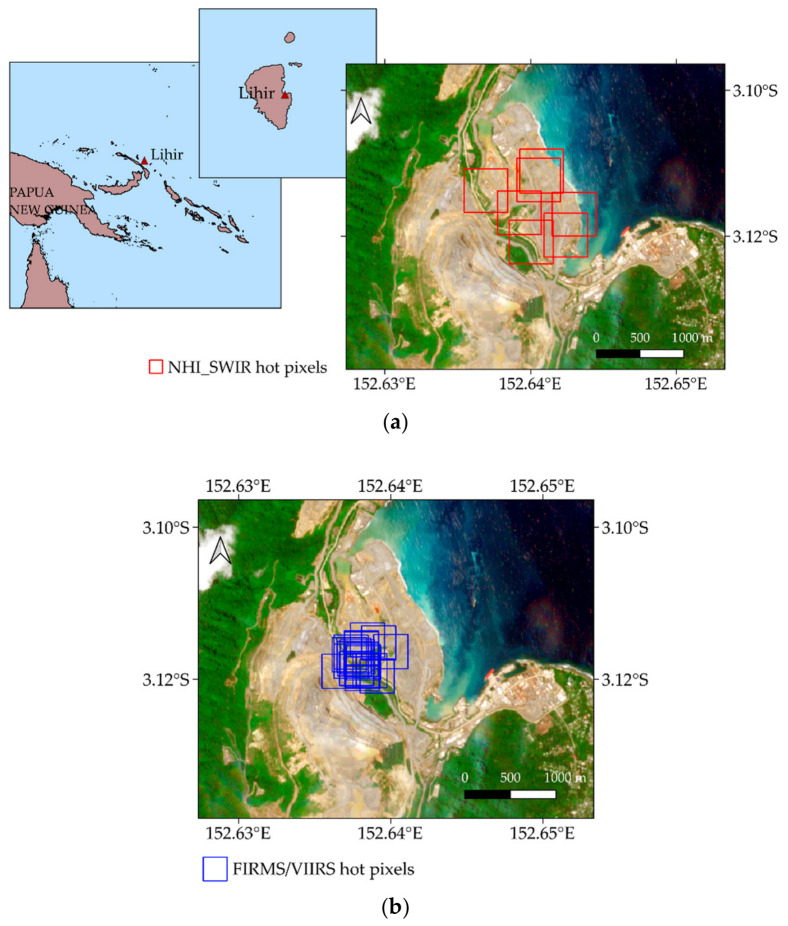
(**a**) NHI-SLSTR detections (in red) performed over the Lihir island, in correspondence of a gold mine located within an active geothermal area; in background the true color image from S2B-MSI data of 1 September 2025 at 00:17 UTC; (**b**) FIRMS detections (in blue) over the same area, from nighttime VIIRS data of 1 August–31 October 2025.

The remaining true hot pixels associated with non-volcanic high-temperature features were flagged over Lihir Island at nighttime, in correspondence with an open-pit gold mine located within a geothermally active zone characterized by hot fumaroles, chloride springs, and hot springs [56] (Figure 6). In this area, high temperatures within the ore bodies (up to 200 °C) may potentially generate hydrothermal eruptions [57]. Therefore, to ensure operational safety, depressurization is primarily achieved through relief wells distributed throughout the area [57].

Even if hotspots detected over Lihir Island (Figure 6a) were not related to geothermal features, which remain undetectable in SWIR bands due to their relatively low temperatures, they were corroborated by FIRMS. The latter detected several thermal anomalies over the island from nighttime VIIRS data of August–October 2025 (Figure 6b). This independent satellite observation confirms the occurrence of transient phenomena, likely high-temperature explosions possibly associated with mining activities.

Hot pixels associated with non-volcanic sources, such as vegetation fires and anthropogenic activities in geothermal areas, increased the percentage of false positives to 3.3% during the first three months of operation. False positives included also pixels erroneously flagged hot by the system due to snow-covered surfaces and SWIR bands co-registration issues (e.g., [58]).

**Figure 7:**
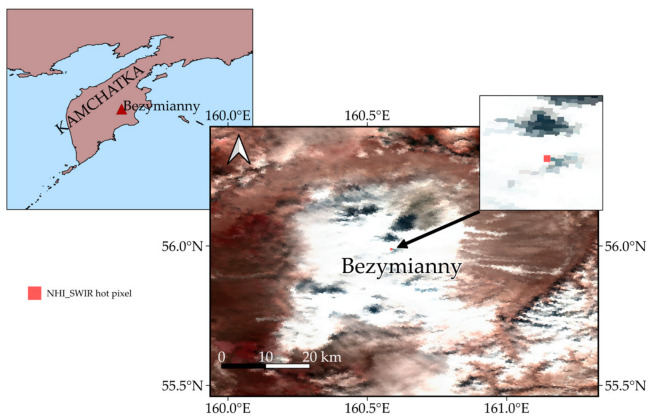
RGB (R = S3, G = S2, B = S1) image from S3-SLSTR overpass of 7 October 2025 at 23:34 UTC, showing the snow-covered Bezymianny volcano (Kamčatka, Russia). The RGB was downloaded from Copernicus Browser. Coastlines are from “GSHHS_f” [50]. In red, on the top-right side of the figure the false detection from the NHI-SLSTR system.

**Figure 8:**
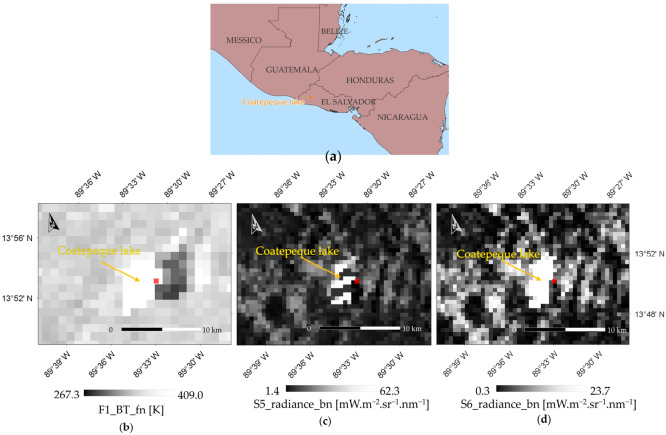
(**a**) Geographical position of the Coatepeque lake, (**b**) MIR band (F1_fn, 3742 nm), (**c**) SWIR S5 band (S5_bn, 1613.4 nm), and (**d**) SWIR S6 band (S6_bn, 2255.7 nm) of the S3-SLSTR image of 4 September 2025 at 16:17 UTC over El Salvador. Red square indicates the hotspot detected close to the crater lake. Coastlines from “GSHHS_f” [50].

Figure 7 shows an example of a false positive from S3-SLSTR data of 7 October 2025 over the Bezymianny volcano because of snow-cover increasing the NHI_SWIR_ index slightly above its critical value. It is worth noting that false positives were usually associated with invalid FRP values because of factors affecting background computation detailed in Section 3.2. This category included also the hot pixels flagged at the margin of Coatepeque (El Salvador) crater lake, a water body that occupies a collapsed caldera, from S3-SLSTR data of 4 September 2025 of 16:17 UTC (see Figure 8). Table A4, in Appendix A, reports values of F1 brightness temperature (F1_BT_fn), S5_bn and S6_bn radiances, and NHI_SWIR_ values for the pixels erroneously flagged as hot due to above-mentioned data issue.

Nevertheless, invalid FRP values characterized even true detections, as observed, for instance, over Kilauea (e.g., from the S3-SLSTR overpass of 3 September 2025 at 20:05 UTC). By considering only hot pixels with positive FRP values (i.e., those showing SWIR radiance above the background), we found that approximately 97.5% of hot pixels were correctly attributed to VTF. A similar accuracy level was maintained when considering the overlap between stripe A and stripe B detections; in these cases, the maximum FRP value (MaxV) within a 300 m spatial buffer was selected (see Table 5).

Furthermore, the classification in Table 6 shows that by filtering out invalid FRP values and pixel duplication, the percentage of false positives decreased to approximately 1.9% (columns 3–5). Although performance of the system should be slightly lower when NRT data are used, this analysis shows that the NHI-SLSTR system identifies volcanic hotspots on a global scale with a low false positive rate.

### 3.2. Preliminary Assessment of FRP Estimates

To assess the FRP estimations from the NHI-SLSTR system, we performed a preliminary comparison with MIROVA by considering only the temporally close satellite observations (temporal gap ≤ 30 min) to minimize the impact of cloudy conditions and time duration of eruptive events on hotspot detection results. Moreover, the comparison focuses only on Etna and Kilauea due to the higher number of concurrent detections. In addition, since SLSTR and VIIRS observations showed a higher temporal gap, we assessed the NHI detections only through comparison with the MODIS dataset. Indeed, the Sentinel-3 satellites equatorial crossing time (i.e., ~10:00 LT and 22:00 LT) is close to that of the Terra satellite [59].

**Figure 9:**
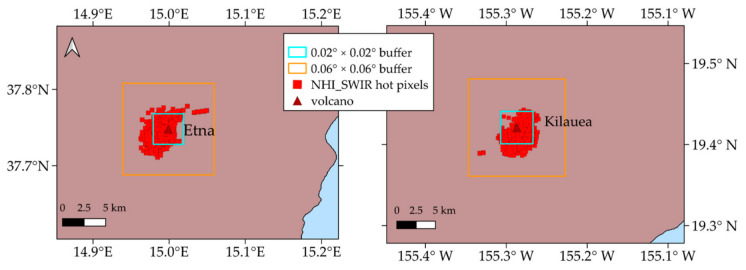
Buffer sizes centered around Etna (Italy) and Kilauea (Hawaii, USA) volcanoes. In red, hot pixels detected by the NHI-SLSTR system over the period of 1 August–31 October 2025. Coastlines from “GSHHS_f” [50].

**Figure 10:**
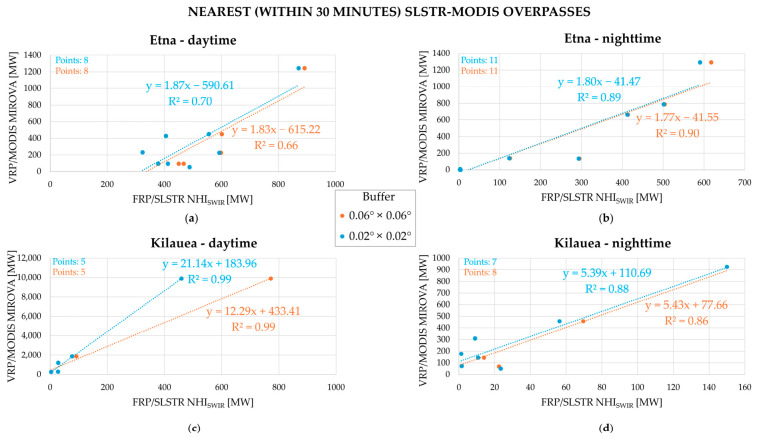
Correlation between FRP-SLSTR (NHI) and VRP-MODIS (MIROVA) performed over Etna (top) and Kilauea (bottom) volcanoes, in reference to the period 1 August–31 October 2025. The FRP was retrieved from SLSTR SWIR data considering two different buffer areas, 0.02° × 0.02° (cyan dots) and 0.06° × 0.06° (orange dots). Plots (**a**,**c**) refer to daytime observations, while plots (**b**,**d**) show the results retrieved in the nighttime conditions.

Several eruptive episodes were recorded at Kilauea since the end of December 2024, with several lava fountaining episodes occurring also during the period August–October 2025 (e.g., [60,61]). To take into consideration these events involving also lava flows in the summit caldera, we considered two different buffer sizes to estimate the radiative power. Specifically, along with the default value, we analyzed a larger buffer area detailed in Figure 9. Figure 10 illustrates the results of this comparison, indicating that, despite the limited dataset resulting from the analysis of few temporally close detections over the three-month period, the FRP-SLSTR (NHI) and VRP-MODIS (MIROVA) estimations were well-correlated.

**Figure 11:**
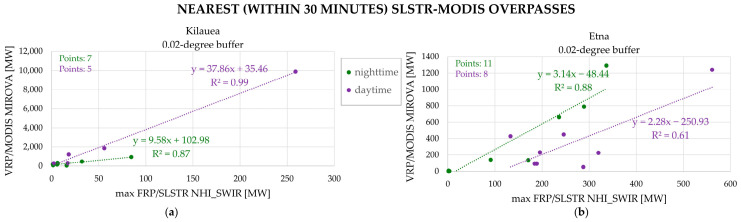
Correlation between FRP-SLSTR (NHI) and VRP-MODIS (MIROVA) performed over Kilauea (**a**) and Etna (**b**) volcanoes, in reference to the period 1 August–31 October 2025, considering a buffer of 0.02° × 0.02° in size. The FRP estimates from SLSTR SWIR data refer to the value retrieved within the buffer after filtering out hot pixel duplication effects (see text for explanation).

The best correlation was observed over Kilauea in daytime regardless of the buffer used. A general good agreement between SLSTR-SWIR and MODIS-MIR observations was recorded also over Etna, especially at nighttime, indicating that both systems provided similar information about variations in thermal volcanic activity. Moreover, when the hot pixel duplication was taken into consideration, the estimates of FRP (SLSTR-SWIR) and VRP (MODIS-MIR) remained highly correlated, especially over Kilauea (Figure 11). The lower agreement was recorded in the daytime over Etna, probably due to a wider range of eruption conditions (i.e., the occurrence also of less intense eruptions).

Nonetheless, the buffer area had a major impact on Kilauea, where meteorological clouds and volcanic plumes affected the daytime FRP computation in a more significant way than Etna.

Although further investigations are required to better assess the estimates of FRP from SWIR data (e.g., extending eruptive periods and analyzing volcanoes with a different lava composition), this preliminary comparison shows the potential of the NHI-SLSTR system in recognizing increasing/decreasing trends of thermal volcanic activity.

**Figure 12:**
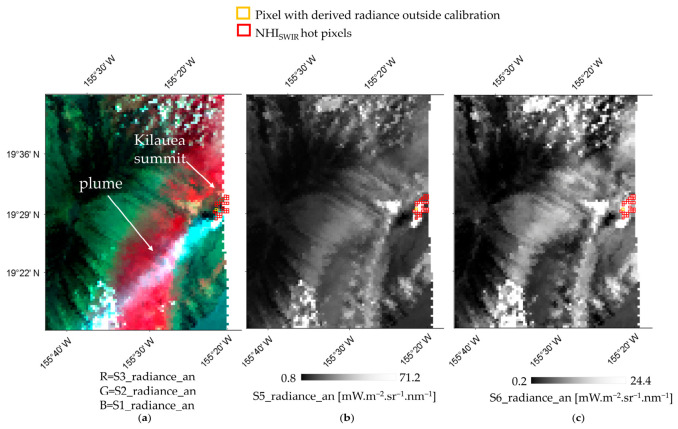
S3-SLSTR data of 19 September 2025 at 20:50 UTC, (**a**) RGB (S3 = 868 nm, 659.47 nm, 554.27 nm) image; (**b**) S5 band (1613.4 nm); (**c**) S6 band (2255.7 nm). Note the volcanic plume (left panel) dispersing in the SW direction from the Kilauea summit caldera, partially affecting the lava flow identification and causing the underestimation of FRP. In red, hot pixels automatically identified by the NHI-SLSTR system.

The RMSE (Root Mean Square Error) values reported in Figure 10 (up to 4300 MW over Kilauea in daytime conditions) indicate, however, that differences in the magnitude of FRP/VRP with MIROVA may be particularly significant even when basaltic lava flows, which should be better characterized through the SWIR retrieval method, are analyzed (Figure 11a). These differences may be attributed to the different sensitivity of the systems to volcanic hotspots as well as to the intrinsic limitations of the above-mentioned method, which tends to underestimate the fire radiative power. A different approach could be then used to retrieve the FRP from SLSTR-SWIR data (e.g., [62]. Differences in the magnitude of FRP/VRP may be then associated also with meteorological clouds and volcanic plumes, affecting the Kilauea’s caldera at the time of SLSTR observations (Figure 12) [61]. These factors caused the drop of the NHI_SWIR_ index below its critical values in daylight conditions, contributing to the FRP underestimation. The significantly lower RMSE values recorded over Etna (Figure 10a,b) and over Kilauea at nighttime (Figure 10d) seem to confirm this evidence. The possible impact of other factors such as the satellite zenith angle (see discussion section in reference to Klyuchevskoy) on estimates of FRP (e.g., invalid radiances and pixel saturation effects) needs to be accurately evaluated.

## 4. Discussion

Results shown in the previous section demonstrate the capacity of the NHI-SLSTR system in detecting and quantifying high-temperature volcanic features, complementing information automatically retrieved in the SWIR bands of MSI and OLI/OLI2 sensors at higher spatial resolution.

**Figure 13:**
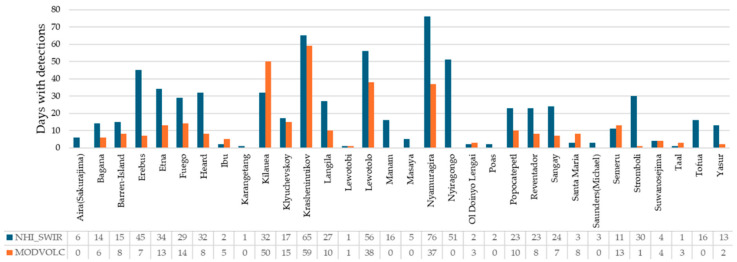
Eruptive days (detailed in the bottom table) from NHI-SLSTR (blue bars) and MODVOLC (orange bards) detections, over the period of 1 August-31 October 2025.

To better assess the contribution of the NHI-SLSTR system to NRT monitoring of active volcanoes, Figure 13 displays the comparison with MODVOLC, performed over the period 1 August–31 October 2025. The NHI-SLSTR system correctly identified hotspots over 32 volcanoes, marking the Nyamuragira (Africa) as the most active, as indicated by the higher number of eruptive days (>70) (see blue bars).

The NHI-SLSTR system then provided unique information about the Aira (Japan), Masaya (Nicaragua), Manam (Papua New Guinea), Karangetang (Indonesia), Poas (Costa Rica), Mount Michael (Saunders Islands), and Tofua (Tonga) activities. Moreover, it provided more continuous information about the Erebus (Antarctica), Heard (Australia), Nyiragongo (Africa) and Stromboli (Italy) activities. Figure 1 also shows that SLSTR SWIR observations may successfully integrate MODIS and VIIRS information, also at the Ol Doinyo Lengai (Tanzania, Africa), which is a unique volcano emitting natrocarbonatite lava, with a temperature around 585 °C [63].

On the other hand, MODVOLC better monitored Kilauea’s eruptive episodes occurring during the investigation period. This difference may be ascribed to the different spectral behavior of degassing/volcanic plumes at 1.6 µm and 2.2 µm wavelengths (e.g., due to ash and/or SO_2_ content), which may cause the drop in the NHI_SWIR_ index value, masking the underlying hotspots. In general, differences with MODVOLC detections may be attributed to variations in the cloudy conditions, time duration of eruptive events, and sensitivity of the systems to volcanic thermal features.

The SLSTR S5/S6 bands could exhibit, in fact, saturation (dynamic ranges of those spectral bands can be found in [64]), such as the S7 band, which is well documented (e.g., [32,33,34]). Indeed, to ensure data quality, saturated or invalid pixels are flagged through the appropriate exception metadata, such as the S6_exception_an (or bn) flags, which distinguish saturation from other types of radiometric anomalies. Hence, if saturation occurs, for instance in the S6 band the NHI_SWIR_ index could decrease below its critical values, causing the underestimation of active lava flows. The latter may be underestimated even when FRP is not computed for all the hot pixels, due to restrictions associated with the background radiance computation.

**Figure 14:**
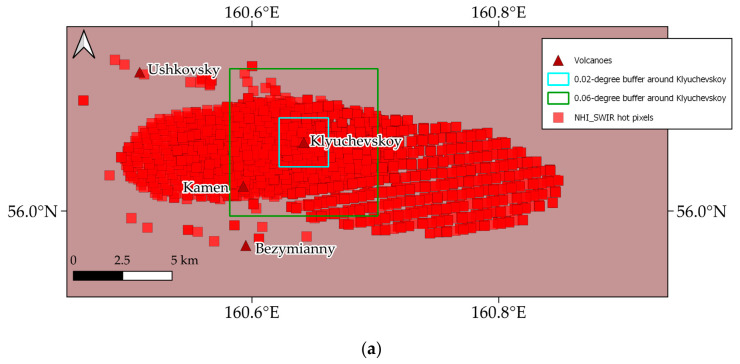

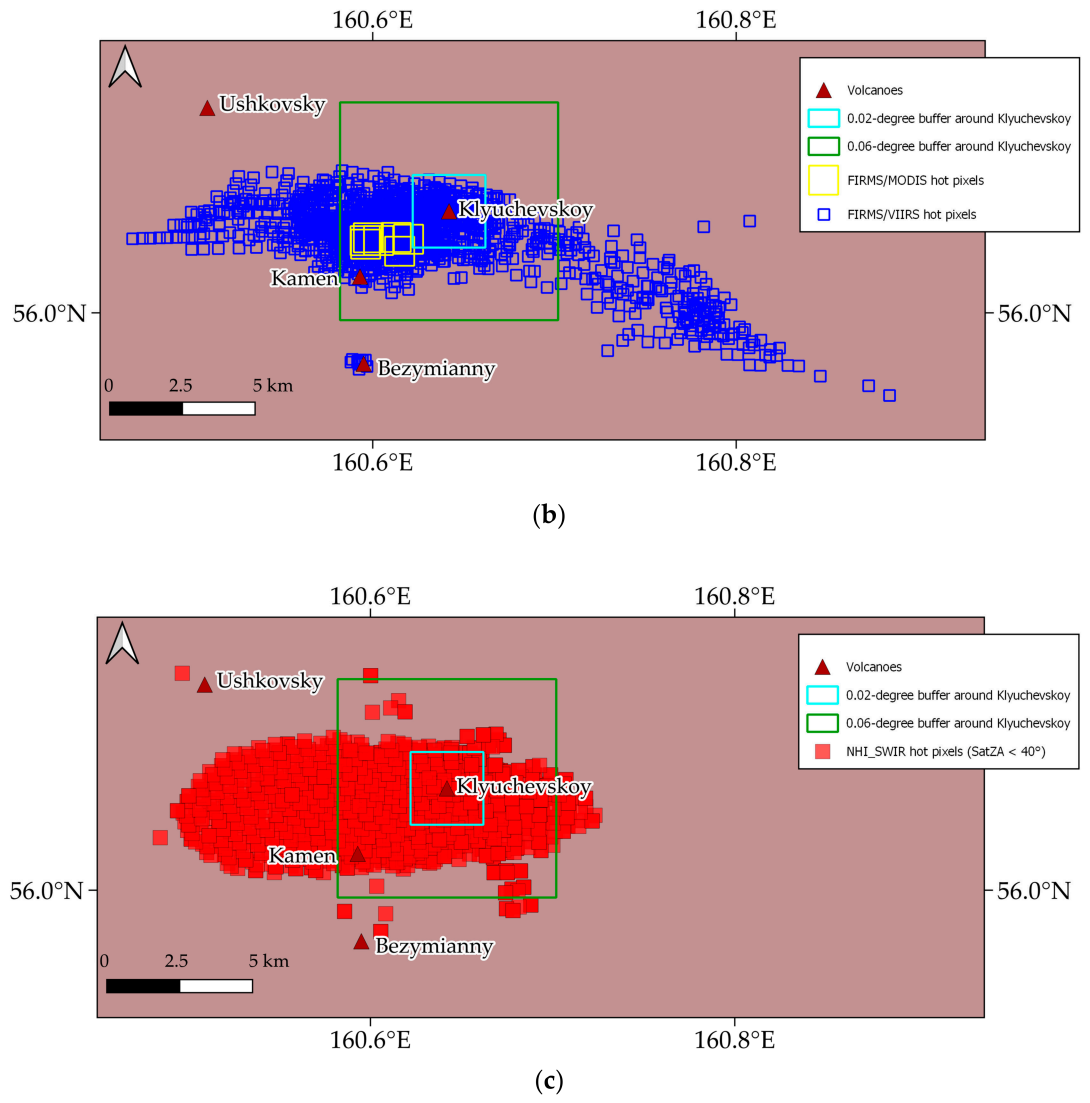
Hot pixels flagged over the Klyuchevskoy (Russia) during 1 August–31 October 2025; (**a**) NHI-SLSTR system (red pixels); (**b**) FIRMS from MODIS (yellow pixels) and VIIRS (blue pixels) data; (**c**) NHI-SLSTR system after filtering pixels with *ϑ_v_* < 40°. Note the reduction of hot pixels detected outside the buffer area (0.06° × 0.06°) marked in green, while the sparsely distributed ones suggest the occurrence of possible data artefacts (e.g., misregistration effects).

Another evident limitation is related to the default buffer area set by the NHI system. If on the one hand the default value minimizes the impact of vegetation fires on volcanic hotspot detection (for some volcanoes such as Vesuvius, the buffer area could be further reduced to cover just the craters), on the other hand, the distal regions of lava flows could remain undetected. A proper setting of the distance buffer remains, however, difficult to perform over some volcanic areas, as shown in Figure 14, in reference to Klyuchevskoy. Indeed, even using a larger buffer area (0.06° × 0.06°), a higher number of hot pixels were excluded from the FRP computation (see red pixels located outside the region marked in green in Figure 14a). The FIRMS detections performed during August–October 2025 confirmed both shape and direction of active lava flows, although the higher spatial resolution of VIIRS observations provided more accurate information about the areas inundated by lava (see blue pixels in Figure 14b).

The hotspot maps also reveal the impact of satellite viewing geometry on the lava flow mapping due to the increase of footprint area towards the swath edges. To take into consideration this effect, the use of a filtering approach based on satellite zenith angle (SatZA) is under evaluation (e.g., note the reduction of hot pixels flagged outside the green box in Figure 14c).

In addition, an improved background computation procedure is currently under testing, since missing FRP values can occur even in the presence of detected hotspots due to an insufficient number of valid background pixels.

Finally, even if the NHI-SLSTR system reports the confidence level for each detected hot pixels, based on snow-presence information retrieved from the 13th bit of ‘confidence_an’ (for stripe A) and ‘confidence_bn’ (for stripe B) files, some hotspots could be mislabeled as low confidence (e.g., over Etna in the winter season). Similar confidence levels could be used to handle false positives associated with vegetation fires. A global vegetation informative layer (e.g., [65]) and/or specific tests on NDVI (Normalized Difference Vegetation Index), computed on daytime SLSTR scenes, could be implemented within the system to flag as “low confidence” hot pixels flagged over vegetated areas.

Despite these limitations, the NHI-SLSTR system remains valuable for monitoring volcanoes, including those located in remote areas for which satellite observations are often the unique source of information (e.g., [30]).

## 5. Conclusions

In this work, we have presented the NHI-SLSTR system for the NRT monitoring of active volcanoes from space. Hotspots detections of August–October 2025 were assessed through comparison with MIROVA, MODVOLC, FIRMS, and the S3 SLSTR L2-FRP products.

The NHI-SLSTR system performs efficient NRT monitoring of high-temperature volcanic features (twice per day over the monitored areas, with an increasing coverage at the high latitudes), despite a certain delay in data processing (<3 h) due to availability in the EUMETSAT data store, thanks to a low false positive rate and despite some limitations mainly associated with meteorological clouds, volcanic plumes, and the default buffer area defined around each monitored volcano. These features may determine the underestimation of detected hotspots, whereas vegetation fires, anthropogenic heat sources, snow-covered surfaces, and SWIR band co-registration issues may lead to false detections. Moreover, because of the spatial resolution of SLSTR SWIR bands, small hot features detected by NHI using MSI and OLI data could be not always identified by the system. This confirms the importance of integrating SWIR observations from different sensors for a more effective investigation and monitoring of high-temperature sources.

In terms of fire radiative power, this parameter may be not always calculated even in the presence of detected hot pixels, due to limitations (insufficient number of valid pixels) of the local background analysis. Moreover, although estimates of FRP from the NHI-SLSTR system show good correlation with those from the MIROVA-MODIS dataset, differences in the magnitude of FRP/VRP can remain significant, as indicated by results retrieved over Etna and Kilauea. Indeed, although the NHI-SLSTR system appears more suited to characterize basaltic lava flows, as their temperature range is closer to that of gas flaring sources than other VTFs, the SWIR retrieval method remains biased toward gas flare emissions. This may lead to significant discrepancies in the magnitude of fire radiative power, especially under specific atmospheric and eruptive conditions (e.g., time duration and intensity of eruptive events). Differences with other systems estimating the FRP/VRP may also arise from limitations in background computation and because of different characteristics of volcanic hotspots (e.g., those having a lower temperature are better detectable in the MIR/TIR bands of MODIS and VIIRS).

Taking into consideration the limitations of the SWIR method, a different approach, previously used to quantify thermal emissions at Kilauea and Mauna Loa (Hawaii, USA) from MSI and OLI data (e.g., [62]) could be applied to Sentinel-3 SLSTR observations. A correcting factor could be also retrieved for harmonizing estimates of FRP/VRP from different sensors having SWIR capabilities.

The system will be further optimized through a more effective background analysis, a better setting of the buffer area, and the implementation of a vegetation layer to better handle fires, with the aim of reducing hotspot attribution errors.

In view of the next system optimization, which will make available also hotspots and FRP time series, this study demonstrates that SLSTR SWIR observations may complement information from MODIS and VIIRS MIR/TIR data, contributing to a more effective worldwide monitoring of active volcanoes from space.

## Figures and Tables

**Table 1:** Main satellite systems performing the global monitoring of thermal volcanic activity in NRT.

Satellite Systems	Sensor	Spectral Bands
MODVOLC	MODIS	MIR, TIR
MIROVA	MODIS/VIIRS/OLI/OLI2/MSI	MIR, TIR, NIR, SWIR
FIRMS	MODIS/VIIRS	MIR
NHI	SLSTR/OLI/OLI2/MSI	NIR, SWIR

**Table 2:** NHI algorithm adapted to SLSTR SWIR data, including a fixed threshold test on S6 radiance used over both land and sea areas. The daytime configuration (DT) unlike the nighttime one (NT) analyses negative critical values of the NHI_SWIR_ index for a sun elevation angle > 5°.

Variable	Threshold Values	
	Land	Sea
NHI_SWIR_	>−0.3 (DT) >0 (NT)	>−0.2 (DT) >0 (NT)
S6 radiance [mW m^−2^ sr^−1^ nm^−1^]	>0.5	

**Table 3:** Number of Sentinel-3 SLSTR imagery, hot pixels flagged by NHI-SLSTR system and detections associated with volcanic thermal features (VTF).

Month	SLSTR Images	Hot Pixels	Hot Pixels Associated to VTF
August 2025	11,877	5696	5234 (91.9%)
September 2025	11,479	3675	3485 (94.8%)
October 2025	11,870	3244	3100 (95.6%)
Total	35,226	12,615	11,819 (93.7%)

**Table 4:** Assessment of NHI-SLSTR detections of 1 August 2025–31 October 2025 performed according to criteria detailed in the text.

True Hot Pixels				False Hot Pixels
Correct Volcanoes Attribution	Uncorrected Volcano Attribution	Fires	Mining Activities in Geothermal Areas	Misregistration Issues and Snow-Covered Surfaces
11,819	381	51	9	355
(93.7%)	(3.0%)	(0.43%)	(0.07%)	(2.8%)

**Table 5:** Number of hot pixels after filtering negative FRP values (NV, column 2 and 4) and selecting pixels with the maximum FRP within a 300 m spatial buffer (MaxV, column 3 and 5) to account for overlapping detections between SLSTR stripes A and B. VTF indicates the percentage of pixels associated with high-temperature volcanic features.

	Total Hot Pixels After Data Filtering		Hot Pixels Associated with VTF After Data Filtering	
	NV [a]	MaxV [b]	NV [c] ([c]/[a] %)	MaxV [d] ([d]/[b] %)
August 2025	4095	2244	3977 (97.1%)	2175 (96.9%)
September 2025	2473	1366	2438 (98.6%)	1341(98.2%)
October 2025	1993	1140	1929 (96.8%)	1096 (96.1%)
Total	8561	4750	8344 (97.5%)	4612 (97.1%)

**Table 6:** Classification of hot pixels (over the period 1 August 2025–31 October 2025) performed by excluding hot pixels associated with invalid FRP values (NV) and considering the maximum FRP (MaxV) retrieved within a 300 m distance buffer to remove hot pixel duplication effects.

	Correct Volcanoes Attribution	Uncorrected Volcano Attribution	Fires	Mining Activities in Geothermal Areas	Misregistration Issues and Snow-Covered Surfaces
NV	8344 (97.5%)	87 (1.0%)	51 (0.6%)	8 (0.1%)	71 (0.8%)
MaxV	4612 (97.1%)	48 (1.0%)	35 (0.7%)	4 (0.1%)	51 (1.1%)

## Data Availability

The SLSTR-NHI web-platform is freely accessible online at https://sites.google.com/view/nhi-tool/sentinel-3 (accessed on 2 April 2026). Data analyzed in this work may be made available under request.

## Abbreviations

The following abbreviations are used in this manuscript:

ASTER	Advanced Spaceborne Thermal Emission and Reflection Radiometer
ECMWF	European Centre for Medium-Range Weather Forecasts
EUMDAC	EUMETSAT Data Access Client
EUMETSAT	European Organization for the Exploitation of Meteorological Satellites
FIRMS	Fire Information for Resource Management System
FRP	Fire Radiative Power
GEE	Google Earth Engine
GF	Gas flaring
GSHHS	Global Self-consistent, Hierarchical, High-resolution Shoreline Database
GVP	Global Volcanism Program
JPSS	Joint Polar Satellite System
KML	Keyhole Markup Language
L8/9	Landsat-8/9
MaxV	Maximum FRP value within a 300 m spatial buffer after removing duplicated hot pixels
MIR	Medium Infrared
MIROVA	Middle Infrared Observations of Volcanic Activity
MODIS	Moderate Resolution Imaging Spectroradiometer
MSI	Multispectral Instrument
NASA	National Aeronautics and Space Administration
NHI	Normalized Hotspot Indices
NIR	Near Infrared
NOAA	National Oceanic and Atmospheric Administration
NPP	National Polar-orbiting Partnership
NRT	Near Real Time
NTC	Non-Time Critical
NV	Negative FRP values filtered
OLI	Operational Land Imager
S2	Sentinel-2
SLSTR	Sea and Land Surface Temperature Radiometer
SWIR	Short-wavelength infrared
SatZA	Satellite zenith angle
TCWV	Total column water vapor
TIR	Thermal Infrared
URP	Urgent Request Protocol
VIIRS	Visible Infrared Imaging Radiometer Suite
VNIR	Visible/Near Infrared
VRP	Volcanic Radiative Power
VTF	Volcanic Thermal Features

## Appendix A. Details of the Hot Pixels Associated with VTF

The 11,819 hot pixels detected by the NHI algorithm and with a correct volcano attribution (see Table 4) have been analyzed in terms of the dominant rock associated to the volcano (Table A1), the corresponding tectonic setting (Table A2) and Country (Table A3).

**Table A1:** Details of the hot pixels associated with VTF, according to the classification of the Smithsonian Institution [40], in terms of the dominant rocks of the volcano.

Dominant Rock Composition	Hot Pixels Associated to VTF
Trachybasalt/Tephrite Basanite	7383
Basalt/Picro-Basalt	2601
Andesite/Basaltic Andesite	776
Foidite	657
Phonolite	307
Trachyandesite/Basaltic Trachyandesite	92
Dacite	3

**Table A2:** Details of the hot pixels associated with VTF, according to the classification of the Smithsonian Institution [40], in terms of the tectonic setting of the volcano.

Tectonic Setting	Hot Pixels Associated to VTF
Rift zone/Continental crust (>25 km)	6778
Subduction zone/Continental crust (>25 km)	3306
Intraplate/Oceanic crust (<15 km)	985
Subduction zone/Crustal thickness unknown	356
Intraplate/Continental crust (>25 km)	307
Subduction zone/Oceanic crust (<15 km)	54
Subduction zone/Intermediate crust (15–25 km)	33

**Table A3:** Details of the hot pixels associated with VTF, according to the classification of the Smithsonian Institution [40], considering the Country of the volcano.

Country	Hot Pixels Associated to VTF
DR Congo	6776
Russia	1509
Italy	1082
United States	713
Indonesia	374
Antarctica	307
Australia	272
Papua New Guinea	175
Ecuador	173
Guatemala	131
Mexico	93
India	89
Vanuatu	33
Tonga	30
Japan	28
Philippines	12
Nicaragua	8
United Kingdom	7
Costa Rica	5
Tanzania	2

**Table A4:** Values of F1 brightness temperature (F1_BT_fn), S5_bn and S6_bn radiances, NHI_SWIR_ values for the pixels erroneously flagged hot (shown in bold) and neighboring ones of Figure 8.

F1_BT_fn [K]			S5_bn [mW·m^−2^·sr^−1^·nm^−1^]			S6_bn [mW·m^−2^·sr^−1^·nm^−1^]			NHI_SWIR__bn		
385.01	331.35	331.35	59.9025	18.4394	18.4394	17.8408	4.65513	4.65513	−0.541	−0.5969	−0.5969
385.01	**331.35**	331.35	5.08043	2.6317	22.8031	3.33372	**1.9729**	5.94804	−0.2076	**−0.1431**	−0.5862
318.88	275.27	275.27	5.08043	1.43866	14.4593	3.33372	0.35718	3.17441	−0.2076	−0.6022	−0.64
